# Optimal Resting-Growth Strategies of Microbial Populations in Fluctuating Environments

**DOI:** 10.1371/journal.pone.0018622

**Published:** 2011-04-15

**Authors:** Nico Geisel, Jose M. G. Vilar, J. Miguel Rubi

**Affiliations:** 1 Departament de Fisica Fonamental, Facultat de Fisica, Universitat de Barcelona, Barcelona, Spain; 2 Lewis-Sigler Institute for Integrative Genomics, Princeton University, Princeton, New Jersey, United States of America; 3 Biophysics Unit (CSIC-UPV/EHU), Department of Biochemistry and Molecular Biology, University of the Basque Country, Bilbao, Spain; 4 IKERBASQUE, Basque Foundation for Science, Bilbao, Spain; Tata Institute of Fundamental Research, India

## Abstract

Bacteria spend most of their lifetime in non-growing states which allow them to survive extended periods of stress and starvation. When environments improve, they must quickly resume growth to maximize their share of limited nutrients. Cells with higher stress resistance often survive longer stress durations at the cost of needing more time to resume growth, a strong disadvantage in competitive environments. Here we analyze the basis of optimal strategies that microorganisms can use to cope with this tradeoff. We explicitly show that the prototypical inverse relation between stress resistance and growth rate can explain much of the different types of behavior observed in stressed microbial populations. Using analytical mathematical methods, we determine the environmental parameters that decide whether cells should remain vegetative upon stress exposure, downregulate their metabolism to an intermediate optimum level, or become dormant. We find that cell-cell variability, or intercellular noise, is consistently beneficial in the presence of extreme environmental fluctuations, and that it provides an efficient population-level mechanism for adaption in a deteriorating environment. Our results reveal key novel aspects of responsive phenotype switching and its role as an adaptive strategy in changing environments.

## Introduction

In their natural habitats unicellular organisms are frequently exposed to stress or starvation and only rarely encounter conditions that allow them to grow. In a competitive environment where growth and stress periods alternate, the species with the largest *time-averaged* growth rate will generally outcompete the others. To achieve this goal, unicellular populations need strategies that both enhance survival during stress and allow rapid resumption of growth as soon as the conditions improve. Controlling these strategies is important for the improvement of biotechnological processing and in the food industry, where microbial survival and regrowth is the main cause of food spoilage [1], [2]. Also the latency times of severe infectious diseases such as cisteriosis, listeriosis and tuberculosis depend on the survival and recovery of microbes, e.g., inside the macrophages. A better understanding of microbial life-strategies may therefore also contribute to the improvement of antibiotic treatments [2]–[4].

The question how a population can maximize its growth in a changing environment is a classic problem in microbiology. Cells can exist in different phenotypes, where each phenotype provides a growth advantage in a particular environment, but a disadvantage in other environments (compared to other phenotypes). Cells can increase long-term fitness by switching between the phenotypes. Previous works have studied the benefits of phenotypic diversity as well as of responsive and stochastic switching between phenotypes [5]–[12]. A central assumption of these works is that the magnitude of the switching rates for a given phenotype can be tuned free from any constraints, and that the transition between phenotypes is instantaneous. Many phenotype transitions, however, take significant time because they involve profound metabolic reorganization and morphological changes, e.g. for starvation survival [13]. A classic example is returning to a fast-growth vegetative state from a non-growing stress-resistant state, which has been observed to take longer the higher the stress resistance [1], [14]–[19]. Here, we envisage a scenario that explicitly accounts for the tradeoff of higher phenotypic fitness in one environment at the cost of longer transition times between phenotypes.

**Figure 1:**
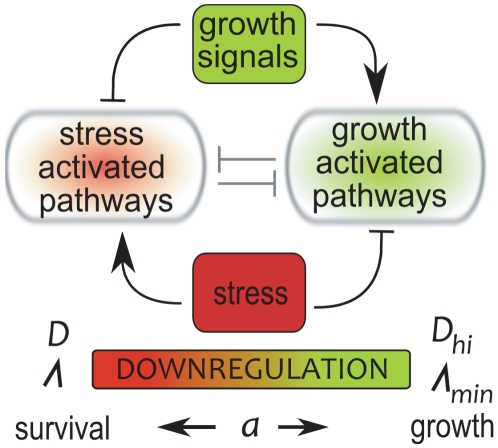
Antagonism of stress-resistance and growth. Growth signals typically repress stress-activated genes and pathways while upregulating growth machinery and growth pathways. Most stress response activators, on the other hand, such as the UspA and MprAB proteins and the SAPK pathway act as growth inhibitors. In most eucaryotes and procaryotes high stress resistance and fast growth are therefore mutually exclusive, and meanwhile cells with high stress-resistance can endure longer stress durations they also have longer reactivation times (growth lags) compared to cells with lower stress resistance (which survive short stress exposure only). We assume that cells which remain vegetative upon stress exposure and do not adapt to stress die at a maximal rate 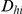, but can quickly resume growth after a short reactivation lag 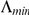 once environmental conditions improve. By downregulating the metabolic activity and entering a stress resistant state, cells can reduce the death rate 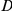 by a factor 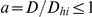, which on the other hand requires them to go through a longer reactivation lag 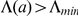 when the environment improves. Thus, 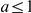 quantifies the tradeoff between stress resistance and growth lag and measures the cellular downregulation during stress exposure.

Adopting a stress resistant phenotype frequently involves growth arrest and the adoption of a metabolically downregulated state [20]–[23]. Maintaining functional growth machinery, such as ribosomes, represents the highest energetic expenditure for stressed cells, which therefore divert their resources towards survival rather than growth when conditions deteriorate, see Fig. 1. Approximately 80% of bacterial biomass resides in such reduced activity states [24] and mutants deficient of such responses rapidly die when exposed to stress [16], [25]–[27]. Downregulated states are thus tremendously important and form an integral part of *microbial* life [22], [23].

In many species stress-induced and growth-induced pathways are antagonists, cf. Fig. 1 [14], [28], hence stress resistance is inversely correlated to growth [18], [19]. Therefore, to restart growth after stress, cells must first re-activate the growth machinery. For starved E.Coli, this process can involve a massive production of ribosomes, from 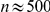 to 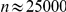, and causes a significant growth retardation with lag-times of up to 20 h [29]. Throughout many species and stressors, this lag time increases with the stress resistance. More specifically, cells able to resume growth quickly (cells with short growth lags) do not survive extended periods of stress, whereas cells surviving prolonged stress periods have significantly longer growth lags. Some examples are *E.Coli*, *L. monocytogenes*, *M. vibrio* and *S. pombe*, after depletion of glucose or nitrogen, exposure to heat stress, freezing, and acidic and salt stress for variable durations. [1], [2], [15], [17].

Thus, when exposed to stress, cells face a tradeoff problem between longer survival and longer growth lags [14], [30]. Highly responsive, most individuals may lose viability by the time nutrient appears. Highly downregulated and resistant, they might resume growth too late, when nutrient has been washed away already or consumed by a competing species [20]. Indeed, long-term evolution experiments have revealed strong selective pressures towards shorter lag phases [30]. The resulting tradeoff is epitomized in the first postulate of microbial ecology: “If you are asleep you won't get dinner” [31].

In the present article we focus on how cells tackle this tradeoff to select optimal strategies for coping with a changing environment. Can populations benefit from delaying a stress response? What determines whether dormancy is a good strategy or not? We consider both homogeneous and heterogeneous populations, taking into account the effects of continuous cell-cell variability, a hallmark of microbial populations under stress. Addressing these questions is important, e.g., for biotechnological processing and treatment of infectious diseases as mentioned above, yet experimental work that can answer them is still sparse [4], [32]. We think our article will stimulate more experimental work: It makes verifiable predictions on the behavior of microbial populations under variable conditions (summarized in the discussion section) and establishes a framework that can guide further experimental investigations. In the discussion section we propose experimental procedures which can verify our predictions.

## Materials and Methods

To understand the implications of the stress-resistance vs. growth-lag tradeoff we propose a model based on the death rates and growth lags of stress resistant and vegetative (active) states. Upon stress exposure cells can enter a stress-protected state, characterized by a reduced death rate 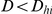 compared to the death rate of the vegetative state 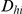 (or of cells unable to respond adequately to stress [16], [25]–[27]). Many species have a short term and a long term stress response which are activated over different time scales [4], [13], [25], [27], [28]. For simplicity we assume that both provide the same stress resistance, i.e., 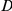 is independent of time.

**Figure 2:**
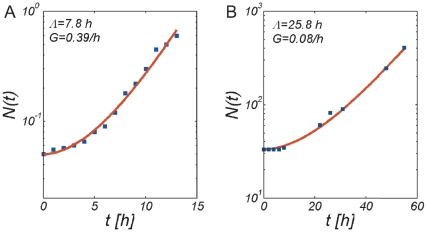
Fit of the growth rate model to experimental growth curves. Values of the fitting parameters 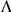 (growth lag) and 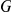 (steady state growth rate in exponential phase) are given in the figures. (A) Batch culture growth kinetics of a mixed bacterial community taken from a biomass recycle reactor after 8 days of starvation. Data taken from Fig. 4B in [33]. (B) Growth curve of *Brochotrix Thermosphacta* after plating on TSA Medium. Data taken from Fig. 1 in [34].



When stress ceases at time 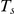, populations start redirecting their resources towards growth. In a growth curve 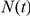 this reactivation appears as a lag phase during which the growth rate increases in time until it reaches a maximal specific growth rate 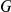, characterizing the exponential phase. This transition can be modeled by a growth rate function 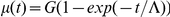 with a lag time 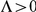. In Fig. 2 we show that this function reproduces experimental growth curves taken from [33], [34], with fitting parameters 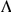 and 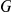. The steady state growth rate 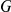 has been shown to be independent of the time 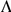 needed to resume growth [29]. We use this equation as a model for recovery and thus can write the growth rate in stress and growth phases of durations 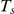 and 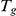, respectively

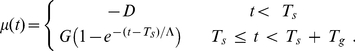
(1)The population size at time 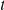 is then obtained from
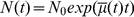
(2)with the time-averaged growth rate 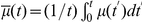. After 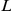 complete cycles of stress exposure and growth (durations 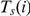 and 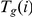), and a total time 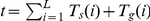 the time-averaged growth rate 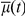 becomes
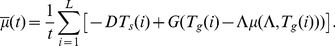
(3)


Here 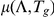 is the growth rate at the end of a growth phase 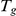. We quantify the tendency of a population to induce stress resistance against the ability to quickly resume growth by the relative reduction of the death rate in the protected state, and define the “activity parameter” 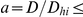
1 (cf. Fig. 1).

The exact dependence of the growth lag on the death rate has not yet been quantified in detail. It is known, however, that the lag time increases with the stress resistance [1], [2], [15], [17], i.e., with 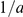 in our model. Expanding this relationship in powers of 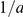 around the vegetative state 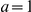 with 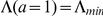 up to linear order, we obtain a first order approximation and can write
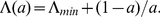
(4)


Hence, the growth lag has a minimum 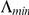 for populations which remain in the vegetative state upon stress exposure (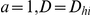), and increases for populations which induce a stress protected state and have higher stress resistance 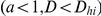. We thus quantify the level of downregulation upon stress exposure by a single parameter 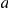.

## Results

### Costs and benefits of downregulation

**Figure 3:**
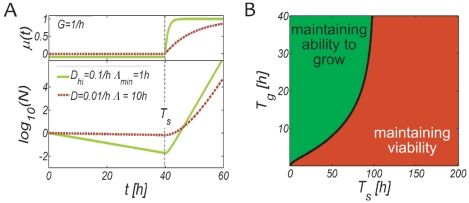
Tradeoffs when adopting stress-protected states. (A) Growth rate 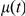 and population size 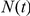 under stress (duration 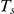) and subsequent regrowth. A population that maintains the active state and remains vegetative upon stress exposure (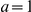, full green line) dies at the maximal rate 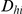 (top panel) and can resume the maximal growth rate 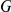 after a minimal growth lag 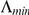 when the environment improves at 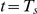. Despite resuming growth with a ten fold lower number of stress-surviving cells, it can outgrow a second population which adopted a stress-protected state (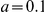, red dashed line) that provides enhanced stress survival 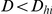 but requires a significantly longer lag time 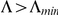. (B) Environmental regimes of stress and growth durations (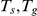) where the stress-resistant (red) or the remaining-active population (green) are more competitive, separated by the black phase boundary 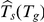. When the typical environment is characterized by frequent but short stress periods, populations can benefit from remaining vegetative upon stress, delaying the protected state, and thereby avoiding growth-retardation after stress. However, the active population also needs a minimal growth duration to reestablish the part of the population that was lost during stress, note the curved phase boundary. Above a maximal stress duration, given by the phase boundary, the loss in viable cells of the vegetative population during stress becomes too large; it cannot reestablish the initial population size before the stress-protected population resumes growth.

To study the tradeoffs when adopting stress-protected states we first consider a single cycle of a stress and a regrowth phase of durations 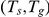 with two homogeneous populations. Figure 3A shows their momentary growth rates and population sizes as obtained from Eqs. 1 and 2. One population (dashed red line) downregulates upon stress exposure into a protected state with typical parameters 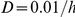 and 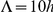 (top panel) [9], [16], [17], [25], [26], [33], [35]. We also consider a population which does not downregulate to avoid the growth lag after stress (full green line) and therefore remains prone to stress. For this strain we assume a death rate 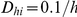 as is the case for starvation-response deficient *E.Coli*, *Vibrio S14*, and *Salmonella typhimurium* mutants, and a lag phase 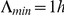 [9], [16], [25], [26]. Most cells of the downregulated strain survive the stress period, whereas a vast majority of the vegetative strain dies (Fig. 3A bottom panel). The survivors of the vegetative strain, however, can quickly resume growth at a high rate (Fig. 3A top panel) as they remained active and maintained an intact growth machinery during stress. For the exposure times considered here (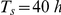, 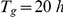) the population that remained vegetative has a higher time-averaged growth rate and outgrows the one which adopted a protected state, despite a ten-fold lower number of stress-surviving cells (note the logarithmic scale).

The population size ratio 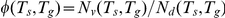 of the vegetative population 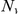 and of the downregulated population 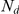 is thus greater than one at the end of the cycle. After 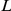 cycles in a time periodic environment of durations (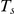,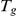) the population size ratio becomes 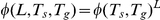, hence differences within one cycle increase exponentially with the number of cycles. To determine the more competitive strategy it is therefore sufficient to consider one cycle only.

To understand which environments favor which strategy (maintaining ability to grow vs. maintaining viability) we calculate the population size ratio 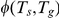 for environmental cycles of different durations (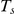,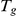), using Eqs. 3 and 2. Figure 3B shows in light green the regime 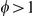 in which the remaining-active strategy is more competitive than the stress-resistant strategy. The black line shows the phase boundary and indicates the maximal stress duration 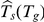 for which the remaining-active strain can outgrow the downregulated one. It is obtained by solving the equation 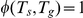 which yields
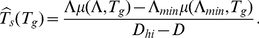
(5)According to Fig. 3B two conditions must be fulfilled for the remaining vegetative strategy to be more competitive: *i)* the stress duration 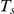 must be sufficiently short such that the difference in stress surviving cells 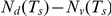 remains small, and *ii)* the growth period 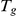 must be sufficiently long such that the active strain can reestablish a large population before the protected strain resumes growth. At very long 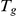 both strains have enough time to reach the exponential growth phase and eventually grow at the same exponential rate 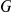. Hence the fraction 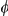 and the phase boundary become independent of 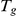. There exists also a maximal stress duration above which the stress-protected population always resumes growth before the vegetative strain reestablishes a comparable population size.

We thus predict that in environments which are characterized by frequent but short stress periods, a population that remains active and eventually delays its stress response can have significant growth benefits compared to populations which rapidly adopt a protected state upon stress exposure.

### Net-growth requires a minimal level of downregulation during stress

**Figure 4:**
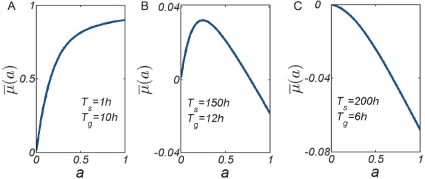
Time-averaged growth rates 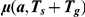 as a function of the downregulation levels 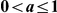 for different environmental cycles of durations (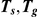). (A) At short stress durations the survival-benefit of a downregulated state (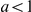) is smaller than the cost of the growth lag after stress. Therefore the time-averaged growth rate decreases with the level of downregulation. (B) At intermediate durations (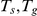) populations which do not sufficiently downregulate (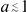) have a negative time-averaged growth rate and go extinct after several environmental cycles. Such populations can increase fitness by adopting a state of higher stress resistance, i.e., by further decreasing 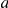. On the other hand, if populations downregulate too much (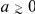) they cannot resume growth sufficiently fast and cannot take advantage of the growth environment. Such populations can enhance their long-term fitness by increasing responsiveness to the improving environment (increasing 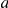, shortening 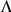), although this results in a lower fitness during stress exposure. (C) At very long stress durations no net growth is possible in the typical environments (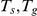). Here the growth benefit which could be obtained during the typical growth period 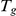, by maintaining the ability to resume growth throughout the stress environment 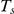, is outweighed by the cost of reduced survival during 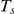.

As we have shown in the previous section, populations which remain in the vegetative state during stress can sometimes outgrow stress-resistant competitors. On the other hand it is clear that such populations will go extinct under sustained stress conditions, i.e., they will have a negative time-averaged growth rate 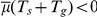. The latter is a measure of fitness in a changing environment [5], [9]–[11] and depends on the death rate during stress and on the lag time during recovery, see Eq. 3, and thereby on the level of downregulation according to Eq. 4. It is likely that unicellular stress response systems have been evolutionary tuned to ensure survival during stress. Thus, we envisage the level of downregulation 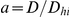 as a variable quantity with 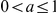, see Fig. 1. Indeed, individual cells within an isogenic population can have very different survival and growth lags [1], [2], [15]–[17], [29]. How much must a population downregulate during stress to not go extinct during the typical cycles of duration (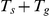)? In Fig. 4 we show how the time-averaged growth rate 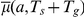 depends on the level of downregulation 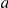 for three different environments 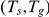. Indeed, positive net growth is not achieved with arbitrary downregulation levels. Instead, as shown in Fig. 4B a population that does not downregulate sufficiently during stress 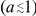 will have a negative time averaged growth rate and can only ensure its survival by further reducing its activity during stress. Thus a minimal level of downregulation 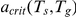 is required to maintain an overall positive growth rate.

**Figure 5:**
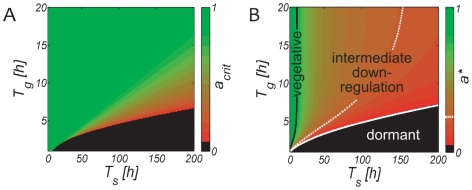
Sufficient and optimal strategies for growth in environments of stress and growth durations (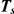, 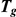). (A) To ensure survival over environmental cycles (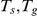) populations must downregulate their death rate by a factor 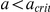 during the stress phases. At long growth and short stress durations a positive time-averaged growth rate can be maintained without adopting a protected state (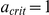). Here the growth benefit during 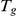 exceeds the death cost during 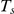 for all levels 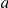. For long stress and short growth durations no net growth is possible because the benefits during growth are outweighed by the costs during the stress phase (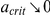). (B) Optimal downregulation levels 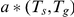 that maximize the time-averaged growth rate 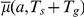. For sufficiently short stress durations 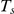 the survival-benefits of stress-protected states are always outweighed by the costs of longer growth-lags after stress. In this regime, limited by the black line, populations need not trade off against survival. The optimal strategy is to remain vegetative upon stress exposure (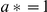). When typical stress durations lay above the black line, populations must reconcile fast recovery with survival. Populations which do not downregulate sufficiently have too large death rates, and eventually go extinct, whereas populations that downregulate too much cannot resume growth sufficiently fast. Such populations can increase long term fitness by decreasing short term fitness, see also Fig. 4B. When the typical growth durations 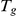 fall below the full white line the optimal strategy is to adopt the state of highest stress resistance, i.e., dormancy, even if this implies to not resume growth during the *typical* growth durations 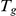. Note that populations with particular downregulation levels are optimal on a *line* in parameter space (see dashed white line on which 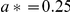).

How does the required downregulation level depend on the environmental conditions? By solving Eq. 3 with 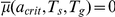 for 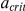 using 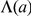 and 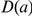 as explained in the model section, we obtain the downregulation level 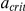 for which the time-averaged growth rate is zero, shown in Figure 5A. All states 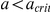 then have a positive time-averaged growth rate. According to Figure 5A, when growth durations are long enough, populations can maintain a positive net growth rate without adopting a protected state during stress (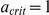 indicated in green). In the opposite regime of very short growth and long stress durations no net growth is possible (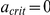 indicated in black). In this regime the cost of maintaining the ability to resume growth during 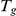 (a larger death rate during 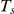) is always greater than the growth benefit that can be obtained during 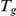. In the intermediate regime, populations with 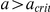 have too little stress resistance and a negative time-averaged growth rate. These populations will eventually go extinct.

To achieve a positive net growth-rate populations must sufficiently downregulate such that the death rate during stress falls below a threshold. We showed that the degree to which stress resistance must be induced not only depends on the conditions of the stress environment but also on the durations of the growth periods.

### Optimal downregulation levels during stress

In natural environments populations must not only survive but rather they must achieve a higher net growth rate than their competitors which means enhancing survival *and* resuming growth faster. As explained previously, the time-averaged growth rate depends on the death rate during stress and on the growth lag after stress. An interesting question to ask is whether there exist optimal induction levels of stress response systems and how these optimal induction levels depend on the characteristics of the microbial habitat. It can be seen already from Fig. 4 that long-term fitness can be maximized by adapting the downregulation level 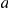. Phrased in the context of our model we thus ask for the optimal downregulation levels 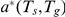 and how they depend on the characteristic environment 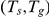
?

To answer this question we solve for the state 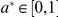 that maximizes 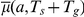 of Eq. 3, i.e. by finding the zeros of 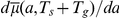. The numerical solutions 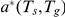 are shown in Fig. 5B and reveal three different regimes of optimality corresponding to the three panels shown in Fig. 4.

In the regime of short stress durations and long growth times, the benefit of enhancing survival during stress is always smaller than the cost of a longer growth lag. Adopting a protected state upon stress exposure reduces the time-averaged growth rate, see Fig. 4A. Hence, in this regime the optimal strategy is to remain vegetative in order to quickly resume growth after a brief stress period.

In the regime of long stress and short growth periods no net growth is possible, as explained in the previous section, see also Fig. 4C. In this regime the optimal strategy is to adopt a dormant state which provides maximal fitness during stress, even if this means to not resume growth during 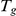 where growth is possible in principle. In this regime net-proliferation is achieved when 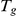 fluctuates to longer than typical values.

In the regime of intermediate stress and growth durations populations must reconcile survival with fast recovery. This is achieved at intermediate downregulation levels a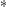, see also Fig. 4B. In this tradeoff-regime suboptimally adapted populations with 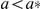 have superior survival during stress, but cannot resume growth sufficiently fast and eventually miss out part of the growth period. These populations can increase fitness by increasing responsiveness 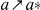, despite reducing the number of stress-surviving cells. Populations with 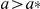 have too large death rates and can increase fitness by increasing survival 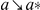, see also Fig. 4B. For very large growth durations 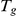 it can be shown that the optimal activity is given by 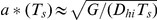, i.e. the optimal stress resistant state becomes independent of 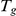 only when 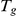 is very large; an optimal population that has reached the steady state growth rate 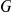 continues to be optimal as growth times increase.

A population is optimal not only in one environment (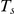,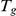) but in a set of environments, indicated by the dashed white line for 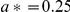 in Fig. 5B. A property which will be important in the context of cell-cell variability, discussed further below.

The inverse relationship between stress-resistance and growth-lags predicts the existence of three optimal strategies where cells would delay their stress response, adopt an intermediate downregulation level or become dormant. Which strategy provides the maximal fitness depends on the typical environmental durations 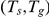. Importantly we found that within the broad regime of intermediate growth durations, the long-term fitness is not maximized by maximizing the momentary fitness in each environment; adopting a highly downregulated state 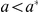 during stress can only provide a short term survival-advantage but does not allow cells to resume growth sufficiently fast. The optimal state during stress exposure therefore also depends on the durations of the growth environment.

### Survival and growth in stochastic environments

**Figure 6:**
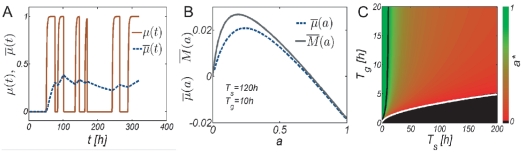
Growth in stochastic environments. Panel (A) shows the momentary growth rate 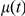 during stress and growth phases (full red line), and the time-averaged growth rate 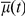 (dashed blue line) which approaches an asymptotic constant 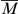 after several environmental cycles. (B) Long-term time-averaged growth rates in periodically 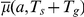 and stochastically 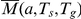 changing environments of mean durations (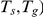 as a function of the downregulation levels. In stochastic environments populations which strongly downregulate (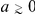) can have a time-averaged growth rate (full gray line) several times higher than in a time-periodic environment (dashed blue line). These differences become negligible when populations recover much faster than the average growth durations, e.g. at (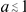). (C) Optimal downregulation levels 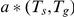 that maximize net-growth 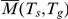 in stochastic environments of mean durations 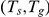. The phase boundaries follow similar lines as in the periodic case.



Although periodic environments are common in nature, more generally the environmental durations 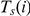, 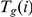 of cycles 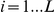 will be random variables. How does this randomness affect our predictions? According to Eq. 3 the time-averaged growth rate 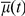 up to a time 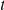 depends on the death rate and lag time as well as the durations 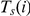 and 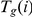. After many environmental cycles 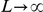, however, the time and fluctuation-averaged growth rate approaches a constant 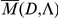, see Fig. 6A. Using the law of large numbers we can replace the summation in Eq. 3 by averages to find

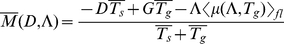
(6)here 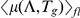 denotes the fluctuation average of 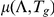 (the growth rate reached by the end of the growth phase) over 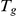; the fluctuation averages of 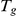 and 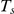 are equal to their time-averages 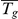, 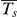. Hence, for 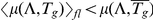 a population will have a higher long-term growth rate in the fluctuating environment of mean duration 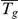 than in a periodic environment of this duration (compare to Eq. 3). Calculating 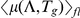 explicitly for an exponential distribution of 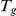 around the average 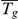 we find
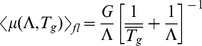
(7)

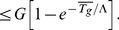
(8)The second line is the value of 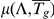 in a periodic environment, see Eq. 3, and the equality follows for 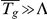. Hence, fluctuations of 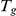 become negligible when 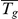 is large compared to the lag time 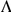. In the opposite case, however, a strain will develop into a larger population in a fluctuating than in a periodic environment, see Fig. 6B. This is astonishing, considering that the exponential distribution has a maximum not at 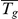 but at 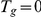, thus the growth period will mostly be shorter than its average. Since growth is exponential, however, a fluctuation towards longer than average durations during the recovery provides a significantly larger benefit than the loss of benefit for shorter than average durations of the same magnitude.

The optimal downregulation levels 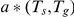 (shown in Fig. 6C) which maximize the long term growth rate can be calculated numerically using 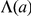 and 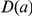 as explained in the model section and from Eqs. 6 and 7. For very long growth durations 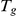, these are identical to 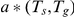 in a periodic environment, compare Figure 5B.

### Effects of cell-cell variability on survival and recovery

Cell-cell variability within an isogenic population has been observed in the stress survival of individual cells [1], [2], [8], [17] and in the single-cell lag times when resuming growth after stress [1], [2], [4], [17], [29], [32]. Stress activated promoters in Yeast are enriched in TATA-boxes and have systematically nosier expression compared to growth activated genes [36]. Also the nuclear shuttling of *Mdm2* appears highly variable from cell to cell. These findings raise the question whether unicellular microbes promote variability during stress rather than suppressing it. Yet it is not obvious what the benefits of intercellular noise could be.

We assume that upon stress exposure the population diversifies into subpopulations of high and low stress resistance, following a gamma distribution 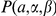 of the downregulation level 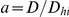 around an average 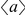, and with parameters 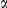 and 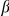. The gamma distribution allows to study symmetric and highly skewed distributions while keeping the average downregulation level constant, and makes the following calculations analytically tractable.

**Figure 7:**
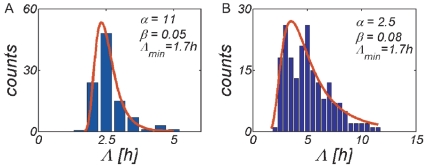
Fits of the lag time distribution 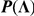 to experimental data. In (A) to the distribution of lag times of *E.Coli* cells resuming growth in LB medium without a foregoing starvation period. Data taken from Fig 4A in [2]. And in (B) to the *E.Coli* lag time distribution after acid stress during 21 days at 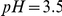. Data taken from Fig. 1D in [2]. Values of the fitting parameters 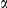, 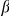 and 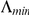 are shown in the figures.

A distribution of downregulation levels results in a distribution of lag times according to the transformation 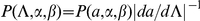, which yields
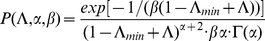
(9)where 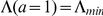. This expression is fitted in Fig. 7 to experimentally measured lag-time distributions with 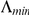,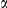 and 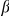 as fitting parameters. The good agreement supports the use of the gamma distribution for the downregulation levels 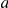.

We measure the (dis-)advantage of intercellular noise by comparing the size of a homogeneous population 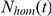 of activity 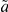, with the size of a heterogeneous population 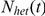 that has the same *population-averaged* stress response 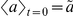 at the onset of stress exposure.

For an average downregulation level 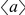 with a standard deviation 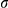 the gamma distribution 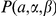 has two parameters given by 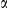 = 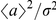 and 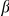 = 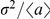 and reads


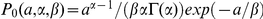
. We consider only values of 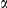, 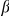 for which the probability of 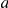 lying outside the interval 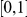 is negligible (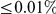). The population size 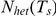 after stress exposure during a time 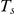 is obtained from the integral
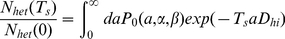
(10)

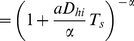
(11)where 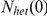 is the initial population size, and 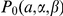 refers to the distribution of 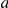 at 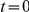 with the average 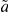. According to Eq. 11, 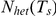 decays algebraically and approaches an exponential decay at small intercellular noise (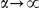).

Importantly, when Eq. 11 provides a reasonably good fit to a colony forming units (CFU) curve under stress, then the fitting parameters 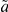 and 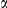 give an estimate of the average death rate and its variability. The distribution of stress resistance in a population thereby becomes readily assessable without the need for single-cell measurements and the generation of histograms.

**Figure 8:**
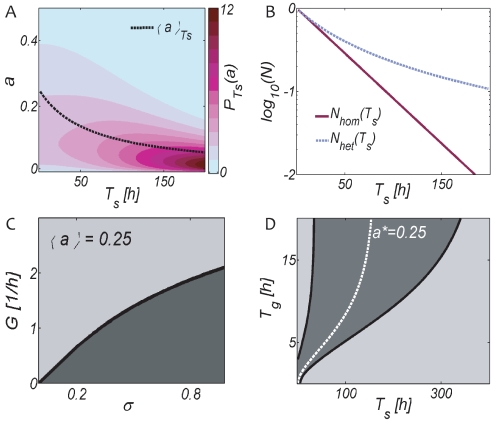
Costs and Benefits of resting state cell-cell variability. (A) Change of the distribution of resting states 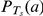 during stress for the initial parameters 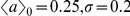. Subpopulations potentially able to resume growth quickly (large 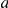) rapidly decline upon stress exposure, resulting in a time dependent average activity (dashed black line) and death rate. (B) The population decay therefore deviates from the exponential decay of a homogeneous population. Panel (C) shows regimes in which cell-cell variability reduces (light gray, 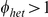) or enhances (dark gray, 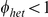) the population growth lag. At large steady state growth rates 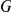, population recovery is driven by the tail of the activity distribution with shorter than average growth lags. At small growth rates 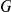, or large variability 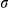, the recovery is driven by the bulk of the distribution with longer than average growth lags. Panel (D) shows regimes of benefits (light gray, 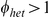) and costs (dark gray, 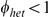) of cell-cell variability in full cycles of stress and regrowth. Heterogeneity represents a disadvantage when the population average is optimally adapted, i.e. when environments are sufficiently periodic and close to the white line compare with Fig. 5B. When environments fluctuate over a wide range, heterogeneous populations benefit from fast responders when the stress duration 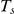 is short, and from highly stress resistant cells when 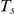 is large.

To understand the resumption of growth of a heterogeneous population we must know the distribution of downregulation levels and lag times by the time 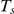, when stress ceases and recovery begins. Normalizing the decaying distribution 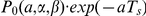 by the total number of surviving cells 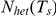, cf. Eq. 11, yields the distribution of downregulation levels 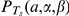 after stress exposure during 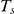


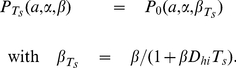
(12)Thus, a population with gamma distributed death rates maintains the gamma distribution, however, with a time dependent scale parameter 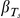. Figure 8A shows how the distribution of downregulation levels 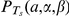 changes while stress prevails for 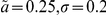. Subpopulations with large death rates 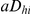 rapidly decline and only subpopulations which have downregulated sufficiently survive. This results in a time-dependent population-averaged activity 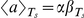 (indicated by the dashed black line) and a time dependent population death rate. Figure 8B shows the resulting algebraic decay 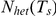 and compares it to the exponential decay of the homogeneous population 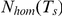.

**Figure 9:**
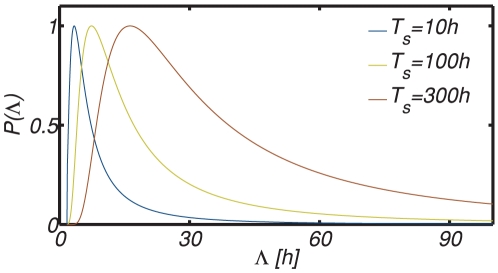
Change of the lag time distribution 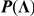 with the duration of stress exposure. Cells able to resume growth quickly do not survive extended periods of stress, hence the distribution 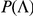 moves to larger values 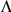. In agreement with the observations in [2] and [29], the most probable value changes only little during the first days of stress, i.e., by a factor of two, whereas the fraction of cells with very long lag times grows significantly as stress prevails. To display all distributions in the same figure, the maximum of each distribution was set to one.

Hence, population heterogeneity of a stress protected state provides a substantial survival benefit at long stress durations. However, as shown in Figure 8A, the survivors are strongly downregulated cells. Therefore, the average lag-time and the tail of the lag time distribution increase significantly with increasing stress exposure time, as observed in [1], [2], [29], [32], [33] and shown in Fig. 9. This may strongly impede the subsequent resumption of growth.

To understand under which conditions population heterogeneity provides a benefit during resumption of growth we must solve the population size equation, Eq. 2, for a distribution of lag times, or of downregulation levels respectively. For one cycle of stress and regrowth it reads:
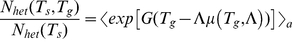
(13)where 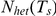 is the population size by the end of the stress period 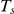 and the average is taken over the distribution of downregulation levels 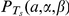 at the time 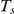, when recovery begins. This integral can only be solved numerically.

It is helpful to first understand the effect of cell-cell variability on the *population growth-lag* only. We therefore set 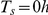 in Eq. 13 and define the fraction 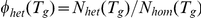 as a measure of fitness. Figure 8C shows the regimes 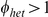 in light gray and 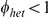 in dark gray as a function of the steady-state growth rate in the exponential phase 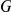, and of the cell cell variability 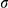, for 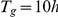 and 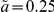. At sufficiently high steady-state growth rates 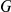, the heterogeneous population benefits from a small but fast recovering subpopulation. The latter can quickly initiate growth, proliferate at a high rate 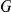, and therefore soon drive the growth of the whole population (tail of the activity-distribution driven recovery). In this case the population growth-lag is shorter than the population-averaged growth lag (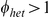, light gray). On the other hand, when 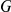 is small, the high activity and fast recovering subpopulations proliferate too slowly to drive population growth. In this case, whole-population recovery does not set in before the bulk of the distribution with longer than average lag times has recovered (the median of 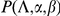 is smaller than its average 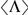 cf. Fig. 9). In this regime the population growth-lag is longer than the population-averaged growth lag, hence 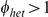 for the bulk driven recovery. Because the distribution is skewed, at increasing variability 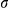 an increasing fraction of the population has lower than average downregulation levels, i.e., longer than average growth lags. To compensate for this, and keep the population growth-lag shorter than average, the decreasing number of fast responding cells needs larger steady-state growth rates 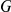. This threshold value 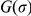 on which 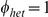 is indicated by the black line in Fig. 8C. Thus, at large 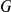 heterogeneous populations can recover faster than homogeneous populations through the tail-of-the-distribution driven recovery mode whereas at small 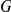 recovery proceeds through a slower bulk-driven recovery mode.

Having considered the heterogeneous population-decline and heterogeneous population growth-lag separately so far, we now ask for the benefits of cell-cell variability in complete cycles of stress and growth 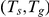, for which we calculate the fitness fraction 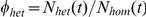 at times 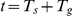 according to Eqs. 2 and 13. Figure 8D shows regimes of beneficial variability (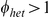) and of disadvantageous variability (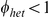) in light gray, or dark gray respectively, for parameters 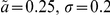. The white line indicates the optimal environments (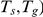 for 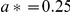, also shown in Figure 5B. Within the dark gray regime, where the population average is sufficiently well adapted, cell-cell variability represents a disadvantage because it decreases the fraction of cells around the optimal state a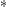. For shorter stress durations 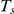, however, the heterogeneous population has a shorter growth lag because fast recovering cells can survive short stress periods and quickly resume the maximal growth rate 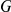. At very long stress durations the heterogeneous strain is more competitive because it contains a number of highly stress resistant cells, see Fig. 8A and 8B. In the regime of short growth and long stress durations, fitness is determined by survival only, because no net-growth is possible on average (cf. white boundary in Fig. 5B). A heterogeneous population always maintains a larger population during stress, hence the lower part of the right phase-boundary in Fig. 8D partly follows the full white line in Fig. 5B.

During stress exposure the distribution of downregulation levels moves to ever decreasing values, due to the death of active and responsive cells. Resting state variability therefore provides a population-level mechanism that progressively sacrifices responsiveness while at the same time increasing stress resistance. This is particularly advantageous in the absence of an energy source, where an energy consuming regulatory mechanism to reliably sense and integrate environmental conditions over time and progressively downregulate individual cells, would represent an additional energetic burden.

We have shown that cell-cell variability always provides a survival-advantage due to the presence of highly downregulated cells. Surprisingly, heterogeneous population recovery can also be slow compared to a homogeneous population if the growth rate after cellular recovery is too small (bulk driven recovery). In environments which are characterized by large fluctuations, intracellular noise appears as a simple strategy to increase the time averaged growth rate, whereas populations should suppress phenotypic variability in deterministically changing environments.

## Discussion

In this article we have studied microbial stress responses as an induced phenotypic switch in stress and growth environments, including the case of cell-cell variability. We have proposed a model which parameterizes a metabolically downregulated state by a single parameter 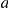. It quantifies the experimentally observed relation between high stress resistance and long growth lags, and allows for a largely analytical treatment. The model reproduces experimental data, makes verifiable predictions, and allows to infer parameters of cell-cell variability from whole-population based measurements such as CFU curves. Our approach provides a framework for experimental investigations and can be generalized to arbitrary functional relationships between stress resistance and growth lag.

We have shown that the inverse relationship between death rate and growth retardation explains and determines in which environmental regimes four commonly observed behaviors of stressed microbes provide a benefit: *i)* Delaying the induction of a stress-protected state, *ii)* adopting a state of intermediate downregulation, *iii)* adopting a dormant state, and *iv)* diversifying the population into cells of high and low stress resistance.

Time delays are a common motif in cellular decision making and frequently appear in microbial stress-responses [37], e.g. in the induction delay of type I persisters in *E.Coli* [4] and the HOG-dependent transcriptional response of yeast to osmotic stress [38]. Our results show that rapidly adopting a downregulated state upon stress exposure may reduce the long term fitness. An optimistic strategy which delays downregulation can provide fitness advantages when organisms frequently face periods of stress exposure lasting less than a critical duration. Precisely this strategy seems to be implemented in *E.Coli*, where the iron stress response is induced only when stress durations exceed a temporal threshold. The delay is mediated by a small non-coding RNA, *IsrR* [39]. Our predictions on the benefit of time delays may be verified in a chemostat of controlled iron-stress and growth durations (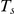,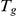) by measuring the time-averaged growth rates of the wild type and *IsrR* knock-out strains used in [39].

In the other extreme, when growth durations are frequently shorter than a limit, a pessimistic strategy becomes optimal: leaving the protected state to resume growth when stress ceases can reduce the long term fitness. In this regime dormancy provides the highest fitness despite the absence of growth in short periods where growth is possible in principle [9], [32]. Here, net growth occurs only during environmental fluctuations in which growth durations are longer than expected. Thus, whether dormancy is a good strategy for survival and growth, not only depends on the stress but also on the growth environment. Importantly, we find that long-term population growth is higher in a stochastic compared to a time-periodic environment. This underlines the importance of periodicity in antibiotic treatments and raises the question for optimal frequencies at which pathogens should be exposed to antibiotics in order to minimize their survival.

Frequently unicellular organisms do not fully shut down their metabolism when facing starvation or stress, but maintain a finite basal activity [13], [15], [16], [24], [25], [27]. Under selective pressures many cellular responses are tuned to optimize certain functions, e.g. the growth rate [5], [40]. An intriguing question is what determines the optimal induction levels of stress response systems. We predict that over a wide regime the optimal metabolic downregulation level during stress is intermediate and determined by the tradeoff between enhancing survival during stress vs. reducing the growth-lag after stress. Optimal downregulation levels thereby depend on the typical durations of the stress *and* growth environments. A suitable model organism to verify this prediction are *Mycobacteria* with externally inducible *mpr-AB* promoters [35]. MprA and MprB activity is necessary for long-term survival, e.g., under amino acid deprivation, but it also represses growth. We propose an experiment in a chemostat, in which an *mpr-AB* inducible strain [35] is exposed to alternating stress and growth conditions, where only the stress environments contain a defined concentration of *mpr-AB* inducer. We predict the existence of an optimal expression (induction) level of the stress response system. Overexpression of *MprAB* can increase the number of stress-surviving cells. These, however, will need too much time to degrade and dilute the growth repressors *MprAB* after stress and to resume the maximal growth rate. On the other hand, suboptimal expression will result in a large population fraction not surviving the stress phase.

These findings have profound consequences for our current view on the role of responsive phenotype switching. When higher phenotypic fitness comes at longer transition times, i.e., when it involves morphological changes and considerable metabolic reorganization [13], [21], [41], the optimal phenotype to induce in an environment only rarely maximizes the fitness in that environment. Instead it must trade off the phenotypic fitness against the transition time and thus it also depends on the frequencies of other environmental conditions, e.g., where the particular phenotype is repressed. This is a novel aspect of adaptation in fluctuating environments which has not been discussed so far. It is also in contrast to the case of *stochastic* switching where fitness is maximized, when the switching rates mimic the environmental frequencies [5], [10], [11].

In heterogeneous populations with intercellular fluctuations of downregulation levels, highly responsive cells rapidly die when exposed to stress, whereas only downregulated subpopulations survive. This results in the prototypic non-exponential decay of colony forming unit (CFU) curves during stress exposure. We have derived an analytical expression which allows inference of cell-cell variability parameters from CFU curves, when the distribution of stress-resistance states in the population is continuous. This is particularly useful because it circumvents the extensive measurements needed to generate histograms and may therefore show great promise for better understanding population survival.

In heterogeneous populations, differential cell death under stress leads to a gradually decreasing population-averaged activity. Thereby the population passively sacrifices responsiveness and increases stress resistance in a deteriorating environment. Such a passive adaptive mechanism which does not require active and energy consuming regulation provides an advantage in the absence of nutrient. An active mechanism which integrates stress conditions over time to progressively downregulate individual cells would represent an additional energetic burden and reduce population fitness.

Previous works found that heterogeneity is advantageous, when individual cells cannot respond sufficiently fast to environmental changes [5], [10], [11]. We have shown that cell-cell variability can also increase the population response time under some conditions, and that it can be disadvantageous when stress durations are intermediate and predictable. In the more general case of irregular environments heterogeneous populations can resume growth more rapidly after brief stress exposure, while better surviving long stress periods. Promoting cell-cell variability therefore appears as a favorable and simple strategy to cope with large environmental fluctuations, which prevail in nature.

Finally we would like to comment on the robustness of our results with respect to the specific details of the model. As yet the exact inverse dependence of the lag-time on the death-rate has not been measured in detail. In this article we have adopted a first order approximation where 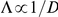. More generally a higher order approximation may be assumed. As long as it is strictly inverse, however, the only change would be a decrease or an increase of the lag-times compared to our first order approximation. This will result in a distortion of the phase diagrams, but would not introduce qualitative changes, e.g., in the general structure of the phase diagrams. We have also performed our analysis using lognormal and normal distributions of death rates and used a sigmoidal recovery function in place of Eq. 1. The results differed in a quantitative way but the conclusions remain unaffected.

Recently many of the molecular players involved in microbial stress responses and cellular downregulation have been identified and the single cell regulatory kinetics have been characterized [4], [32], [35], [38], [39], [42], [43]. In the present article we have provided a first approach to quantify the ecological consequences of the stress-resistance vs. growth constraints, which we hope will stimulate more experimental work.
